# BIAS AND GENERALIZABILITY IN MEDICARE-BASED ANALYSES ACROSS THREE POLICY PERIODS

**DOI:** 10.1093/geroni/igad104.1988

**Published:** 2023-12-21

**Authors:** Galina Gorbunova, Arseniy Yashkin, Julia Kravchenko, Anatoliy Yashin, Igor Akushevich, Eric Stallard

**Affiliations:** Duke University, Durham, North Carolina, United States; Duke University, Durham, North Carolina, United States; Duke University School of Medicine, Durham, North Carolina, United States; Social Science Research Institute, Duke University, Durham, North Carolina, United States; Duke University, Durham, North Carolina, United States; Duke University, Durham, North Carolina, United States

## Abstract

Administrative Big Health data such as that drawn from the Medicare program is an invaluable resource for the study of population health. Although the Medicare program provides near universal health coverage for older U.S. adults age 65+, for most types of care data is collected only for individuals enrolled in traditional Medicare (TM) plans, with those enrolled in private Medicare Advantage (MA) plans being effectively censored in terms of the extent of available data. This presents two potential problems for health outcomes research: bias and generalizability. In this study we use Health and Retirement Study data (1998-2015) to assess the presence and magnitude of group-specific differences between MA and TM beneficiaries as well as plan switchers across three policy-specific periods (Balanced Budget Act; Medicare Modernization Act; Affordable Care Act). We found that the MA population was characterized by higher diversity, adverse behavioral habits, lower education, and economic disadvantage. Initially, lower morbidity levels converged with the TM average over time. Individuals, switching into MA after TM had much larger morbidity levels and mortality risk than any other Medicare group. We found significant loss of generalizability with respect to the Hispanic population; such estimates could be further biased to the extent that MA Hispanics differ from TM Hispanics. Of great concern is the combination of low education and low economic status in MA beneficiaries – a combination also found in many disadvantaged minorities. Care is needed in the design of TM-based studies, especially those focused on ethnic differences.

